# Veterinary Surgery

**Published:** 1891-06

**Authors:** S. E. Weber


### REVIEWS.

"*Tierarztliche Chirurgie, fur Praktische und Studierende.*"

VETERINARY SURGERY FOR PRACTICAL VETERINARIANS AND STUDENTS. BY L. HOFFMAN, PROFESSOR AT THE VETERINARY SCHOOL, IN STUTTGART. PUBLISHED BY SCHICKHARD AND EBNER. PARTS I. II. III. IV.

A hiatus in Veterinary Science, especially in America, has been that of a good practical work upon Surgery of the Domestic Animals, one which, it might be said, was on a par with the best modern works on human Surgery. As the author is one of the noted German Clinicians, we greet with great pleasure the announcement of the publication of this work and rest with the hope and confidence that a great want is being filled.

This book does not appear as a complete volume, but in parts of which we have received, four. The material is divided in two parts, the first of which is devoted to *special* and the second, to *general surgery;* under the *special part* we find the diseases of the *head*, each being placed in its proper organic category, and most elaborately treated. There could be no better arrangement of the subject matter of this part.

The author omits all introductory remarks, and starts out with the work by giving the localization of brain function, and diagnosis of diseases of the same, with fig. 1, showing the different functional centers. Each topic treated upon is accompanied with a bibliography, giving reference to extensive literature on the subject.

Under the head of Diseases of the Nose and Throat, we find illustrated a most valuable instrument (the *Rhino-Laryngoskope*), by the use of which a thorough diagnosis of the parts is made possible. The first instrument made use of for this purpose in Veterinary Science was by Polansky and and Schindelka in 1889, but since then the instrument has been wonderfully improved and is now a most intricate piece of mechanism, though in appearance a simple tube with a bulb at its end. By its optical arrangement it is possible to see the most trivial steak on the mucous membrane reflected and highly magnified on the glass in the bulb on the outside. Through this great advancement it has been made easy to diagnose diseases heretofore very difficult. It is invaluable in the diagnosis of inflammatory diseases of the throat, glanders, compression of the larynx, roaring, etc.

The second part is almost wholly devoted to diseases of the *Eye*, and the able manner in which the author deals with the surgical affections of this organ bespeaks great experience.

There are 640 pages in the four parts of the work before us, with 100 illustrations, mostly original, and with a very few exceptions the cuts are as fine as it is possible to execute them on wood. We are unable to point out a single word of criticism in the diseases of the *thorax* and *abdomen*, which part is put forth in a very thorough manner, and the intelligent arrangement of diseases of the *bowels*, with special stress to those of the *rectum* shows the author's good judgment. It is impossible (in a description of this nature) to give the part of the work before us, the justice it demands, and and we hope the author will finish the other parts of this grand work in keeping with the same standard, and thus usher into Veterinary Science a work which will be a monument to himself and a pleasure to the veterinarian.

The following is a translation of the author's Prospectus of the work :—

"Since the scientific reformation of surgery, the triumphs of which consists in antiseptics, arresting of hemorrhage, and anaesthesia as well as in microscopic diagnosis, there has not so much been done in the production of veterinary literature, as could have been expected, in comparison with human surgery, and corresponding to the importance of the subject.

Having become thoroughly intimate with the principals and workings of modern surgery, I have, in preparing the book before us, brought throughout, the new method, wherever my experience saw advantage in its application.

I have, however, through literary studies, gained the conviction, that the former, or now past surgery contains such a rich treasure of knowledge and experience, that the modern doctrines appear as some noble, mighty Scion grafted on the tree of former surgery. But as a green limb is only able to live when it remains in communication with the tree, so can, according to my opinion, modern surgery produce the most perfect results only through him who is perfectly acquainted with its issues. The knowledge and observations of our predecessors, appear to me as necessary for that purpose, as the tree is necessary for the vitality of the branch. For this reason I have made special and detailed researches into the literature. The practical object was my leading motive.

To govern the extensive matter in a regular way, I have divided the work into a *Special* and *General* part. The special part will appear first, so as to give immediately an idea in what way the real practice has been treated.

The Publishers deemed it best to publish the book in parts, of which it is proposed to issue from nine to ten, each of about ten forms. The manuscript is so far prepared, that the book will be finished within one year."

S. E. Weber.

